# Professional identity formation for underrepresented groups in medicine: challenges and interventions for Dutch medical schools: a systematic scoping review

**DOI:** 10.1186/s12909-025-07811-6

**Published:** 2025-12-26

**Authors:** Meka Abdi, Olga Kanzaki Sooudi, Megan Milota

**Affiliations:** 1 Honors Medical Program, Utrecht University, Utrecht, The Netherlands; 2 Department of Anthropology, University of Amsterdam, Amsterdam, The Netherlands; 3 Julius Center for Health Sciences and Primary Care, UMC Utrecht, Utrecht, The Netherlands

**Keywords:** Professional identity formation, Medical students, URiM students, Diversity, Equity, Inclusion, Medical schools

## Abstract

**Background:**

The concept of intersectionality is important when considering the professional identity formation (PIF) of students who are racially and ethnically underrepresented in medicine (URiM), as they must navigate race and ethnicity within the medical landscape. Despite a growing body of studies that shed light on the challenges that URiM students face in their PIF, there remains a notable lack of practical interventions for medical schools to address these challenges. Our objective is to highlight the challenges faced by URiM students and identify interventions in the literature that would be most suitable for Dutch medical schools to address them.

**Methods:**

This study builds upon Teo et al.‘s (2022) scoping review. We examined articles from January 1, 2000, to December 31, 2021, and conducted an extended search from January 1, 2022, to November 30, 2023. Our focus was on articles exploring the intersectionality of PIF, perspectives of minoritized groups, and diversity, equity, and inclusion (DEI) within the context of PIF in medical education. We used the Systematic Evidence-Based Approach (SEBA) guided systematic scoping review, encompassing four stages: Systematic Approach, Structured Summary and Synthesis, Jigsaw Perspective, and Literature Analysis.

**Results:**

A total of 692 abstracts were reviewed, 36 full-text articles were evaluated, and 22 articles were included. URiM students encounter multiple challenges in their PIF journeys such as a lack of role models and representation, experiences of microaggressions, and pressure to assimilate into the majority culture. The proposed interventions for medical schools included diversifying recruitment practices to create more role models, developing curricula to address these challenges, and establishing a supportive network for URiM students.

**Conclusions:**

Our study highlights the pressing need for Dutch medical schools to address the challenges faced by URiM students in PIF. The identified interventions offer actionable strategies to cultivate a more supportive and equitable learning environment. The implementation of these interventions has the potential to enhance URiM students’ educational experiences, reduce disparities, and promote diversity within the medical profession. These findings underscore the importance of ongoing efforts to prioritize inclusivity and equity in medical education.

**Supplementary Information:**

The online version contains supplementary material available at 10.1186/s12909-025-07811-6.

## Background

In many Global North contexts, the healthcare sector faces persistent underrepresentation issues. The term Global North is not a geographical designation, rather it refers to economically and technologically developed, wealthy nations and thus includes countries in North America, Western Europe, as well as Australia and some parts of Asia [1]. Many of these nations also have ethnically diverse populations due to histories linked to colonialism and/or more recent waves of labor migration, yet this societal diversity is not necessarily reflected in the medical profession. For instance, in the United States only 7% of physicians and 12.5% of physician assistants belong to underrepresented in medicine (URiM) groups [2]. Until the mid-19th century, Black students were systematically excluded from most U.S. medical schools, with only a few institutions in the North and East admitting a limited number of Black students [3]. Once they graduated, Black doctors were confined to segregated healthcare settings, where they exclusively cared for Black patients and assumed subordinate roles to White physicians [4]. Following 1865, seven historically Black medical colleges were established [3]. However, the implementation of Abraham Flexner’s 1910 medical education reforms led to the closure of all but two of these institutions [5], further restricting opportunities for Black students and perpetuating racial disparities in medical education and the physician workforce.

In the Netherlands, the pressing concern of underrepresentation is similarly evident, as the medical specialist population fails to reflect the diversity observed in both the general population and among medical students [6]. For instance, only 2–4% of medical specialists have a migration background, meaning they have at least one parent who was born outside the Netherlands, compared to 21% of medical students. Additionally, Turkish-Dutch and Moroccan-Dutch individuals, who constitute 4.8% of the population, represent just 1.6% of physicians and 1.2% of specialists [6]. These disparities highlight a ‘leaky pipeline’ effect in the transition from medical education to professional practice, further limiting opportunities for URiM students to find relatable role models within the profession [6]. 

Further, professional identity formation (PIF) is a critical process for medical students, involving the internalization of norms, values, and behaviors necessary to “think, act, and feel like a physician” [7]. This developmental process is influenced by interactions with peers, mentors, and the broader medical community [7–9]. However, URiM students—defined as those racial and ethnic populations underrepresented in the medical profession relative to their numbers in the general population [10]—could face challenges that can hinder PIF.

Dutch medical schools face many of the same problems related to URiM students as described in studies originating in other countries. However, certain cultural and historical factors unique to the Netherlands bear consideration. While the Netherlands is an ethnically diverse country (25% of Dutch residents were first- or second-generation migrants as of 2022) [11], it is only in recent years that many societal discussions around race, institutional racism and discrimination against minoritized groups, and related issues like decolonization have begun. What is perhaps distinctive about the Dutch context is what anthropologist Gloria Wekker has described as “White innocence,” which refers to how hegemonic, White Dutch identities are built on self-images of innocence and goodness, of being a highly tolerant, civilized nation that champions human rights, while at the same time invisibilizing national colonial histories and the experiences of post-colonial (Surinamese and Indonesian) and migrant (Turkish, Moroccan, and others) populations [12]. In other words, racialized white identities are coded in highly positive moral terms, while those racialized as non-white are not.

In the Dutch medical field, normative whiteness underpins the “cultural cloning” of physicians [13]. Attempts are being made to publicly address historical and contemporary sources of inequity. For example, the Dutch king recently acknowledged and apologized for the Netherlands’ historical role in the colonial slave trade [14]. In addition, diversity, equity, and inclusion offices have been established in many public organizations like universities and in the private sector in the last few years. At the same time, a recent government report showed widespread individual experiences of discrimination as related to cultural and other forms of difference [15], and in the medical profession in particular, minoritized ethnic groups are systematically extremely underrepresented [6, 16]. At the national level, a recent dramatic rise in rightwing populist rhetoric that stigmatizes immigrants and fuels anxieties about cultural integration has culminated in the right-wing party, Party for Freedom (PVV) becoming the largest political faction in government since 2023 [17, 18]. Thus identifying actionable strategies to support the professional identity formation of URiM students is of both urgent institutional and public importance.

In addition to being underrepresented, students with ‘non-Western migrant’^1^backgrounds at all Dutch universities have higher dropout rates and slower study progress than their ethnic Dutch peers [19]. The higher dropout rates among students from minoritized groups may be attributed to a lack of adequate role models and experiences of active discrimination [20–22]. These challenges could potentially impact the PIF of current Dutch URiM students. Studies indicate that the concept of intersectionality plays a crucial role in PIF, as students navigate the intersection of race and ethnicity within the medical landscape [23]. The term ‘intersectionality,’ coined by law scholar Kimberlé Crenshaw, describes how multiple elements of an individual’s social identity, such as race, class, and gender, interact in real-time to create and perpetuate societal inequalities and discrimination [24]. 

Krishna’s Ring Theory of Personhood expands on this by examining how societal, cultural, and professional expectations shape medical students’ identities [25]. It highlights that conflicts between these external expectations and a student’s personal or cultural identities can lead to “disharmony” or even “dyssynchrony” [25]. To address these tensions, students may employ strategies like “patching,” where they adopt an ‘ideal’ identity to bridge gaps in their professional identity, or “splinting,” relying on past identities to protect their current, fragile sense of self. This contextual understanding becomes crucial when considering the challenges confronting URiM students in their PIF.

An increasing number of students from ‘non-Western migrant’ backgrounds are enrolling in Dutch medical schools, with the percentage of first-generation students growing from 2.4% in 2018 to 3.1% in 2023, and that of second-generation students increasing from 7.2% in 2018 to 9.4% in 2023 [26]. The rise in enrollment of minoritized students in Dutch medical schools signifies progress towards a more diverse medical workforce, ensuring that medicine more accurately represents and reflects the patient body it serves. Simultaneously, this growth emphasizes the need for Dutch medical schools to address the challenges faced by URiM students in their PIF and to support them accordingly. Additionally, the discourse surrounding DEI within Dutch medical schools shows that normalization practices in academic hospitals often obscure diversity and hinder the inclusion of culturally minoritized professionals [27]. These practices frame professionalism as neutral and equate equality with sameness, perpetuating an unequal distribution of privilege and disadvantage among professionals. Such cultural norms and organizational structures contribute to entrenching the difficulties URiM students face in their PIF.

Despite the growing body of studies exploring these challenges, there remains a notable lack of practical interventions for addressing them. Consequently, our systematic scoping review aims to provide valuable insights for Dutch medical schools on how to best support these students. Our primary research question for this study is therefore: ‘In what ways can Dutch medical schools support URiM students in their PIF?’ We started by outlining the challenges encountered by URiM students, followed by an analysis of interventions proposed for medical schools to address these challenges. Additionally, we explored whether these interventions primarily target minoritized groups or address normative practices within the institutions. While we use the case of Dutch medical schools as our empirical focus, given the widespread nature of underrepresentation as a problem in diverse healthcare contexts, and the centrality of PIF to the professionalization of all physicians, our findings can be relevant for scholars and medical educators in other countries where URiM are present.

## Methods

This systematic scoping review builds upon the foundational work of Teo et al. (2022), which explored how cultural, religious, and societal influences shape the complex process of PIF in medical students [25]. Teo et al.‘s insights provided a valuable starting point for addressing the challenges faced by URIM groups. Additionally, Teo et al.‘s expansive coverage of the articles from 2000 to 2021 ensures a broad spectrum of literature for our analysis. Their inclusion and exclusion criteria were designed to identify studies relevant to PIF among medical students. Our study built upon their methodology but refined it by adding a critical criterion: all selected articles were required to explicitly address DEI, intersectionality, or provide meaningful insights into the experiences of minoritized groups within the context of PIF. Articles were sourced globally to ensure a comprehensive understanding of underrepresentation and diversity in medical education.

Teo et al.’s inclusion criteria focused on studies conducted within undergraduate and postgraduate medical schools, specifically examining methods of assessing PIF. Articles were included if they analyzed principles, modalities, or criteria for PIF assessment or investigated the impact of such assessments on medical students. A broad range of study designs was eligible, including mixed methods research, meta-analyses, systematic reviews, randomized controlled trials, cohort studies, case-control studies, cross-sectional studies, and descriptive papers. Eligible studies had to be written in English or translated into English and published between January 1, 2000, and December 31, 2021. The databases searched included PubMed, Embase, ERIC and Scopus. Teo et al.’s exclusion criteria ruled out studies that focused on qualified physicians (including residents) without direct reference to medical schools. Research related to allied health fields, such as pharmacy, dietetics, or physiotherapy, was also excluded unless explicitly linked to medical schools. Similarly, studies on non-medical specialties, such as veterinary science, dentistry, or alternative medicine, were excluded unless they addressed PIF within medical schools. Case reports, editorials, conference abstracts, and opinion pieces were also excluded.

We recognized a scarcity of articles within Teo et al.’s included studies that align with our inclusion criteria. To enhance our approach, we conducted backward and forward snowballing on the included articles, but this process yielded no relevant articles. To address the scarcity of articles specifically centered on the intersectionality of PIF, DEI, and URiM perspectives, we therefore conducted an extended search from January 1, 2022, to November 30, 2023, using the same search string employed by Teo et al. [25]. This focus allowed us to assess how aspects of diversity are integrated or overlooked in the PIF literature for medical students. Our search yielded 1,001 articles from four databases: PubMed (287), Embase (356), Scopus (288), and ERIC (70). After removing 309 duplicates, we screened 692 records by title and abstract for relevance to DEI, URiM populations, and PIF. Following the application of exclusion criteria, 656 studies were excluded. Next, 36 articles underwent full-text review. Of these, 23 were excluded based on the inclusion and exclusion criteria, leaving 13 eligible studies. Additionally, to ensure comprehensiveness, backward and forward snowballing was conducted for these 13 studies, identifying nine more articles. This process resulted in a total of 22 articles for detailed analysis.

**Fig. 1:**
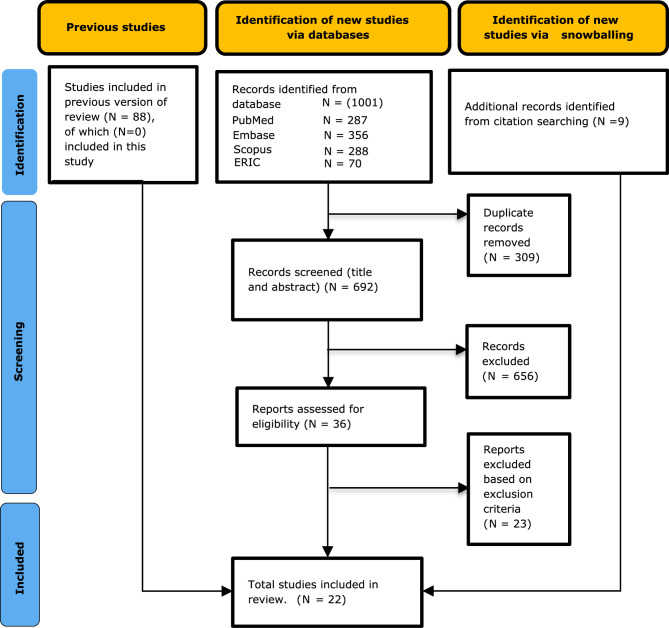
PRISMA Flowchart

Figure 1 presents the PRISMA flowchart summarizing the screening and selection process. For transparency and reproducibility, the detailed search strategy is provided in Appendix A, and a tabulated summary of the 22 included articles is available in Appendix B.

### SEBA approach

To ensure methodological consistency, we adopted the Systematic Evidence-Based Approach (SEBA) for our systematic scoping review, following the methodology outlined by Teo et al. [25]. We followed the four stages of SEBA as outlined by the authors and provide a description below of how this approach was applied to our research question.

#### Stage 1: systematic approach

In Stage 1 of SEBA, we embraced a systematic approach to formulate the research questions and establish inclusion criteria. Our primary research questions centered on how Dutch medical schools can support URIM students in their PIF. Secondary questions examined the specific challenges faced by URiM students and whether the interventions primarily target minoritized groups or address normative institutional practices. This structured foundation ensured clarity and coherence in our inquiry.

#### Stage 2: structured summary and synthesis

During stage 2, we conducted an in-depth analysis of full-text articles, creating tabulated summaries according to established standards (Appendix B). This process was essential for evaluating the selected articles comprehensively and organizing findings systematically.

#### Stage 3: Jigsaw perspective

In stage 3, the Jigsaw perspective was used to integrate insights from various sources that contributed to a comprehensive understanding of PIF. Initially, we synthesized insights from the first workshop for the “Disrupting Sameness in Dutch Academia” consortium project, which served as a catalyst for our engagement in diversity and inclusion efforts within Dutch academic medicine. This project investigates how individuals and organizations perpetuate privilege and marginalization through maintaining norms, and aims to develop interventions that disrupt these patterns in Dutch universities. The workshop provided valuable insights into the challenges and opportunities in promoting diversity and inclusion within the medical field.

Additionally, we conducted focus groups with participants from various groups within the medical community, including medical students from the Student Diversity and Inclusion platform at the University Medical Center Utrecht, year representatives of medical students at Utrecht University, bachelor’s and master’s medical students, and physicians. This approach enabled us to comprehensively map out the key themes and perspectives within the medical community regarding diversity and inclusion.

#### Stage 4: literature analysis

In Stage 4, we analyzed the data-driven literature by synthesizing recurring themes related to the challenges faced by URiM students in their PIF and potential interventions within medical education. Building on the structured summaries created in Stage 2 (Appendix B), this stage focused on identifying and grouping common themes and patterns across the included studies. The findings of this synthesis are detailed in the Results section.

## Results

URiM groups not only encounter obstacles in their journey toward becoming medical specialists [6] but also encounter a myriad of challenges in their PIF. In the following section, we delineate these challenges, addressing issues such as a lack of role models and representation, experiences of microaggressions, and pressure to assimilate into the majority culture. Appendix B further illustrates the information derived from the included studies.

### Challenges

#### Lack of role models and minority tax

Insufficient role models and mentorship present a challenge for URiM groups, with URiM 196 groups expressing a longing for mentors. [21, 28, 29] In studies, URiM professionals reported 197 feeling the need to act as positive role models for others with minoritized backgrounds due to a 198 lack of role models for themselves. This mentoring activity can be characterized as participating 199 in ‘racial uplift’. [2, 30, 31]Racial uplift is a double-sided process. On the one hand, URiM 200 professionals integrate their racial and professional identities by giving back to their 201 communities of origin for the support they received in pursuing educational and professional 202 goals; [30] on the other hand, URiM professionals are expected to present and advocate for their 203 communities, constituting an additional burden, described as the ‘minority tax’ [32]. 

#### Microaggressions

Another challenge faced by URiM groups is the experience of multiple forms of microaggressions from various sources, including fellow students, faculty, guest lecturers, and administrators [2, 20]. They describe feelings of heightened visibility due to their skin color and encounters with patients who refuse their care based on racial bias, thereby highlighting pervasive discrimination within healthcare [20]. Additionally, participants discussed the added pressure of having to work twice as hard because they felt scrutinized more stringently due to the color of their skin and a stronger sense of being under a microscope in comparison with their White counterparts [20]. These instances illustrate the stress URiM students face as they balance professional learning with the imperative to prove their worth in a demanding environment, emphasizing the profound impact of microaggressions. Most notably, microaggressions can lead to depressive symptoms which can ultimately hinder academic advancement in medicine [33]. 

#### Pressure to assimilate into the majority culture

URiM groups face pressure to assimilate into the majority culture, leading them to adjust their behavior accordingly [2, 34]. For example, in one study, a Black orthopedic surgery student revealed a constant need to suppress expressions of anger to avoid conforming to the stereotype of the ‘angry Black woman’ [34]. 

URiM groups also experience pressure to conform to Eurocentric beauty standards [35]. This aligns with broader scholarly studies demonstrating that individuals, particularly Black women, may internalize European beauty ideals [37]. Similarly, certain Black medical students adopt strategic measures, such as employing a ring light, to present a lighter complexion during residency interviews, which exemplifies the effects of colorism [35]. These observations align with the concept of ‘double consciousness,’ wherein students with minoritized identities navigate society with a dual awareness [36]. URiM physicians may grapple with an internal conflict due to ‘double consciousness’—that is, belonging to the medical in-group while not conforming to the prototypical identity of a physician [38]. 

### Interventions

The existing challenges emphasize the need for medical schools to support URiM groups. In the following section, we explore the literature to identify proposed interventions for medical schools to address these challenges.

#### Increasing diverse role models

Studies mention that increased representation of diverse role models is essential for fostering an inclusive and supportive environment [2, 39]. Mentorship strengthens the in-group cohesion of medical students and contributes to identity-safe environments [38]. It can also contribute to a strong sense of belonging, which can in turn lead to more students staying in college and graduating [40]. 

To achieve the presence of more diverse role models, studies suggest various approaches. These include diversifying recruitment practices and intentionally recruiting individuals from URiM backgrounds to invite them into the profession [2, 39]. This can enhance URiM students’ sense of support, mentorship, and belonging within the medical school community [39], and help create an environment that promotes academic development psychological safety [2].

The I-CA^2^R^2^E framework, originally developed to support medical students in their PIF, outlines strategies such as fostering individual connections, acknowledging students’ experiences, adjusting education to be more inclusive, and role modeling [41]. One study noted the framework’s potential for addressing the unique challenges faced by URiM students [29]. 

Studies additionally suggest that minoritized professionals should have more access to leadership positions within academic medicine [30]. Compared to their non-URiM counterparts, URiM faculty members express greater aspirations for leadership in academic medicine; however, they experience lower levels of inclusion, trust, and relationships within the academic environment. To address the leadership aspirations of URiM physicians, it is crucial to focus on the leadership ambitions of this group. These leadership opportunities should encompass positions at the institutional level [30]. 

#### Sharing narratives of lived experiences

Another proposed intervention in medical education is to make room to share lived experience narratives with students. Banks’s theory of multicultural education highlights the positive significance of integrating a curriculum that reflects the lived experiences of minoritized individuals and is grounded in equity pedagogy [42]. In the educational context, narratives based on lived experiences can be leveraged to deepen understanding, cultivate empathy, and enhance cultural competence [42]. One suggested intervention in medical education includes incorporating a professionalism module focused on sharing experiential narratives into the curriculum [43]. In this module, physicians, including those from underrepresented groups, can be invited to recount career-defining moments that necessitated reconciling personal and professional identities. The presence of URiM physicians could contribute to a heightened sense of belonging and an increased sense of representation for URiM students.

#### Elevating awareness about socialization

Studies indicate that the socialization process in medical education can create pressure for URiM students to assimilate into the dominant culture, which may conflict with their existing values and beliefs [44]. 

Research also highlights that PIF is influenced by values and ontological systems cultivated during childhood, suggesting that students should be given more time to reflect on their diverse perspectives and backgrounds during their medical training [44]. Faculty-student interactions, such as small-group sessions or one-on-one meetings, are identified as opportunities to better understand students’ values, beliefs, and practices shaped by their upbringing and communities [44, 45]. 

#### Discussing privilege and underprivilege in medical education

Moreover, studies emphasize the importance of addressing socioeconomic experiences, privilege, and underprivilege in medical education to address disparities among medical students [44]. Doing so can contribute to an environment where medical students from underprivileged backgrounds feel more easily seen and understood by their peers and educators. Additionally, it broadens medical students’ understanding of patients from disadvantaged backgrounds, which could shape their PIF. To facilitate discussions on sensitive topics such as socioeconomic experiences and privilege, educators are encouraged to create a safe space for medical students to engage in these conversations. This involves remaining attentive to inadvertent microaggressions that may arise during these discussions [44, 46]. 

#### Fostering identity safety

Lastly, it is also acknowledged that minoritized individuals play an active role in cultivating a sense of identity safety for themselves by addressing the challenges that they face [21]. Minoritized individuals contribute proactively to their psychological and educational well-being. They engage in behaviors such as seeking mentors with similar identities, engaging with leadership for accommodations, and fostering a sense of belonging, all of which contribute to this proactive sense of safety [21]. Additionally, studies highlight the positive impact of diverse identities, which are viewed not only as challenges but as assets that enhance patient care through effective communication and unique perspectives [21]. 

Out of the 22 articles included in this study, only six presented concrete approaches to address the challenges faced in PIF. Table 1 provides a comprehensive overview of these articles, summarizing key interventions proposed in the literature to address challenges in PIF for URiM groups [2, 30, 31, 39, 43, 45]. 

**Table 1:** Overview of the proposed interventions for medical schools

Authors/Year	Type of study	Study group	Proposed interventions
Frost, 2013 [45]	Critical literature review	Medical education	Faculty should develop a personal understanding of students by engaging in explicit conversations about identity construction, clarify goals, provide small group and one-on-one interactions to shepherd students through their PIF.
Wyatt, 2020 [31]	Qualitative interviews	Black/African American medical students (*N* = 14)	Faculty need to develop robust training programs for non-URiM physicians to learn how to mentor URiM students, in this way, the minority tax on URiM physicians to guide URiM students will be alleviated.
Wyatt, 2021 [30]	Qualitative interviews	Black/African American medical students (*N* = 14), residents (*N* = 10), physicians (*N* = 17)	URiM trainees should have more access to leadership positions within academic medicine.
Bhatia-Lin, 2021 [43]	Descriptive	Medical education	Medical schools should implement a professionalism module in which URiM physicians are invited to share lived experiences and narratives with the medical students to guide them in their PIF.
Wooten, 2023 [2]	Qualitative Interviews	URiM PA students and PAs (*N* = 45)	To increase mentorship, institutions should recruit racially and ethnically minoritized individuals. Schools should also intentionally reach out to minoritized communities and bring students into their programs.
Nemiroff, 2024 [39]	Qualitative Interviews	URiM students (*N* = 13) and non-URiM students (*N* = 21)	Medical schools need to diversify recruitment practices. Diversifying the student body and hospital workforce could bolster URiM students’ sense of support, mentorship, and belonging within the medical school community.

### Consideration of normative practices vs. minoritized groups

As we delve into the interventions proposed for URiM groups in their PIF, the following question arises: Do these interventions target norm groups (and thereby target normative practices within medical institutions) or do they focus on minoritized groups? In the following section, we will first elucidate the normative practices and the norm group within our context, and after that, categorize the findings into these two domains.

#### Normative practices and the norm group

Normative practices refer to established customs, behaviors, or procedures within an institution that reflect dominant cultural norms or values [47]. Historically, normative practices in medical schools included segregationist policies that excluded Blacks/African Americans from medical education [3]. Contemporary normative practices within medical schools may encompass the belief that norms of medical professionalism are deeply rooted in the historical and Eurocentric portrayal of a physician, leading to instances where URiM groups report being scrutinized or singled out based on their cultural or ethnic attributes [2, 35]. 

The norm group comprises individuals who adhere to the dominant cultural norms or values within a given context. For example, medical students in Dutch medical schools are disproportionately likely to have parents with a very high income [48]. In 2020, 56.3% of first-year medical students had at least one parent within the top 10% highest-income earners in the Netherlands. This shows that the norm group among Dutch medical students primarily consists of individuals from higher socioeconomic backgrounds [48]. 

#### Interventions for norm group and minoritized groups

Several interventions identified in this study targeted both the norm group and minoritized groups. For instance, adapting medical curricula to incorporate diverse narratives, including sharing the lived experiences of minoritized individuals, benefits both URiM students and students from the norm group [42, 43]. By introducing diverse narratives into the curriculum, this approach disrupts existing normative practices that may perpetuate exclusivity and bias.

Additionally, some interventions focus on educating medical educators about the socialization process in medical education. Studies indicate that this process can create pressure for URiM students to assimilate into the dominant culture, which may conflict with their existing values and beliefs [44]. By promoting awareness of this socialization process, educators can help students navigate the tension between their cultural identity and the expectations of the medical profession. In doing so, these interventions seek to disrupt normative practices that perpetuate a singular, often homogeneous view of the medical profession. The I-CA^2^R^2^E framework addresses normative practices, the needs of minoritized groups, as well as the norm group [29, 41]. By encouraging self-awareness and reflection among faculty members, it challenges existing norms. Simultaneously, it could provide essential role models for URiM students, enhancing their sense of belonging. Additionally, it fosters individual connections and open exchanges, creating supportive relationships that directly benefit minoritized individuals in predominantly White institutions.

#### Interventions that primarily focus on minoritized groups

Fostering identity safety tends to focus primarily on individuals from minoritized groups. This intervention suggests that URiM individuals should actively contribute to their well-being by seeking mentors with similar identities, engaging with leadership for support, and fostering a sense of belonging, which can enhance their identity safety [21]. Another intervention focuses on the recruitment and leadership opportunities for URiM faculty. Actively recruiting URiM faculty and providing access to leadership positions can make medical schools a more inclusive environment [30]. This approach focuses on supporting minoritized groups by providing resources to them specifically, rather than addressing normative practices. However, it is noted that recruitment alone may not be sufficient to bring about lasting changes in normative practices, and broader strategies targeting institutional policies and culture may be needed for enduring impact.

## Discussion

### Findings

Our study investigates the challenges encountered by URiM groups in their PIF and explores interventions proposed in the literature that aim to address these challenges. We found that there has been a recent shift in the scholarly landscape regarding research on PIF in URiM groups. Notably, a substantial number of studies have been published since 2022, indicating increased scholarly interest and recognition of the importance of addressing the challenges faced by URiM groups in PIF. This shift may be indicative of a growing awareness within the academic community about the significance of DEI in medical education.

Below, we discuss these challenges, structured around three key themes: lack of role models, microaggressions, and pressure to assimilate into the majority culture. We also consider the appropriateness of the suggestions in the included studies and consider their implications for Dutch medical schools.

### Challenges

#### Lack of role models and minority tax

Insufficient role models and mentorship remain a challenge for URiM groups, who often express a longing for mentors with similar identities [29]. This need for role models is strongly felt by Dutch URiM students as well [28]. Dutch URiM alumni have expressed that having more doctors from minority backgrounds would have been beneficial for them [28]. Many URiM professionals engage in racial uplift by taking on mentoring roles to support others and give back to their communities [30]. Additionally, they experience the burden of the ‘minority tax’ [32].

#### Microaggressions

Microaggressions remain a pervasive challenge for URiM students with reports of discriminatory interactions involving peers, faculty, and patients [2, 20]. Many students describe heightened visibility due to their racial or ethnic identity, often encountering discriminatory experiences such as patients refusing care based on racial bias [20]. This heightened scrutiny exacerbates the pressure to perform exceptionally, as URiM students feel pressured to demonstrate their competence more than their White counterparts [20]. Such persistent pressure can adversely impact the academic and professional success of URiM students [33]. 

In the Netherlands, similar patterns emerge among Dutch URiM students. Many report feeling discriminated against based on their ethnic, cultural, and/or religious identity, particularly in specific parts of the curriculum such as group discussions on biases, case studies, and practical training sessions [49]. In these situations, Dutch URiM students often remain quiet to avoid further scrutiny [50]. 

Moreover, a qualitative interview study of female Muslim medical students in the Netherlands reported that certain characteristics, such as wearing a headscarf or speaking with a foreign accent, were associated with being perceived as ‘different’ and contributed to feelings of exclusion [50]. Respondents reported microaggressions, like being told they couldn’t be ‘neutral’ because of their headscarf [50]. Such tensions underscore the challenges URiM students face in reconciling their personal and professional identities in a medical environment.

#### Pressure to assimilate into the majority culture

Studies report that URiM groups face substantial pressure to assimilate into the majority culture to align with Eurocentric standards of professionalism [34, 35]. This is evident in behaviors such as suppressing emotional expressions to avoid conforming to racial stereotypes, such as the “angry Black woman” [35]. Furthermore, some Black students strategically alter their appearance, including using ring lights to present a lighter complexion during residency interviews, underscoring the pervasive effects of colorism [35, 37, 51]. 

Professional appearance norms further perpetuate stereotype threats, as seen in white coat ceremonies where directives on ethnic hairstyles, such as keeping them “neat,” implicitly suggest these styles are less professional [2]. These norms reflect societal biases, positioning Eurocentric features as ideal while framing cultural attributes as deviations. This dynamic forces URiM students to navigate “double consciousness,” balancing their cultural identity with the expectations of the medical profession, complicating their PIF [38]. Although these findings are results related to American medical educational settings, it is possible that Dutch URiM students encounter similar pressures. As a minority in medical environments, they may experience comparable biases and societal expectations regarding professional appearance.

### Putting interventions into practice

#### Increasing representation

To address these challenges, interventions for Dutch medical schools should prioritize the increase of diverse role models.

Studies suggest that it is not sufficient for institutions to wait for racially minoritized individuals to express interest in the medical profession. Instead, schools should actively reach out to these communities and invite them into their programs [2]. Although URiM physicians often engage in racial uplift by mentoring URiM students, this responsibility should not rest solely on them [31]. A study recommends that this burden be shared among all medical educators, transitioning from a minority tax to a collective responsibility [30]. To facilitate this shift, academic institutions should implement robust training programs for non-URiM physicians to prepare them for mentoring URiM students [31]. These programs can help mentors understand the sociohistorical context of URiM students, enabling effective support in integrating their racial and professional identities.

#### Increasing awareness about socialization

Studies indicate that the socialization process in medical education often pressures URiM students to assimilate into the dominant culture, conflicting with their existing values and beliefs [44]. Medical educators should critically reevaluate this process and move away from attempting to mold students into a predetermined image of the “right kind of doctor” [44]. Instead, educators should focus on teaching students about their socialization process, acknowledging that PIF is influenced by childhood values and broader ontological systems [44]. 

Additionally, educators should strive to understand students’ values, beliefs, and practices shaped by their home communities. This can be achieved through explicit conversations about identity construction, clarifying goals, and providing small-group or one-on-one interactions to guide students on their professional journeys [45]. 

#### Fostering identity safety

Minoritized individuals play an active role in cultivating a sense of identity safety by addressing the challenges they face [21]. They proactively contribute to their psychological and educational well-being by seeking mentors with similar identities and fostering a sense of belonging. Shifting the focus from external factors to individual agency highlights the empowering role of diverse identities in creating a supportive and safe learning environment [21]. 

### Implications for Dutch medical schools

Dutch medical schools must address the challenges faced by URiM students, as studies reveal significant systemic barriers among Dutch URiM students. When considering the applicability of the proposed interventions to Dutch medical schools, it is essential to assess their feasibility and effectiveness within the local educational and societal framework. The I-CA^2^R^2^E framework provides a structured approach to guide these efforts for fostering inclusivity in medical education, as many of the proposed interventions align well with its components.

Dutch medical schools should aim to establish a connection between mentors and Dutch URiM students. Given the large student populations in Dutch medical schools, scalable approaches, such as small-group mentoring or peer-support networks, may offer a feasible solution. Additionally, Dutch medical schools should enhance role model representation through inclusive recruitment practices. By prioritizing competence-based recruitment and addressing systemic biases, institutions can ensure diverse representation that reflects the patient demographics in Dutch society. Furthermore, it is crucial to create safe spaces for open dialogue, acknowledge and validate student experiences, and adapt teaching methods to foster inclusivity. Adjusting expectations and curricula can further contribute to a more equitable learning environment. By integrating these strategies, Dutch medical schools can address systemic barriers faced by URiM students, fostering an inclusive educational environment that supports their PIF and contributes to a more equitable medical profession in the Netherlands.

### Limitations

This study had several limitations. First, a substantial portion of the articles included were qualitative studies which could impact the generalizability of this study. Second, this study acknowledges a surge in literature from 2022 onward. This temporal bias may limit the historical perspective on URiM students’ professional identity challenges and interventions. Third, the study emphasizes the need for increased representation of diverse role models as a key intervention. However, the effectiveness of this strategy may depend on various factors, such as the quality of mentorship and the specific contexts in which role models interact with URiM students. Finally, the study discusses proposed interventions without delving into the implementation process or potential barriers to their execution. Challenges in translating these interventions into practice may exist, and future research should explore the feasibility and effectiveness of these strategies in diverse medical education settings.

### Strengths

Despite the limitations of the study, its numerous strengths contribute valuable insights to the discourse on PIF for URiM groups. One notable strength lies in its holistic approach, as the study delves into various facets of URiM individuals’ professional identity, encompassing challenges, interventions, and the broader institutional and societal context. Furthermore, the study’s recommendations and interventions bridge a crucial gap in the current literature by providing actions for educators, institutions, and policymakers. Therefore, this study adds a practical dimension to the theoretical insights, providing actionable steps to address the identified challenges in the PIF of URiM groups. Additionally, this study aligns with broader societal goals of promoting DEI in medical education. Its focus on URiM groups contributes to advancing DEI initiatives within the medical field. In summary, these strengths collectively position this study as a valuable resource, providing both theoretical insights and actionable recommendations for transformative changes in medical education practices.

### Further research

This study establishes a foundation for comprehending these challenges and proposing interventions in PIF for URiM groups. However, to deepen our understanding and guide future interventions effectively, several avenues for further research have been identified.

One such avenue is exploring the practical incorporation of the proposed interventions, particularly assessing their feasibility, challenges, and effectiveness within medical institutions. Such research could inform evidence-based strategies to promote inclusivity in medical education. Another key area for further exploration is the inclusivity of recruitment and hiring procedures within medical institutions. Investigating how these processes ensure diverse representation and equitable opportunities for underrepresented groups will provide insights into how institutional change can be fostered.

Furthermore, the qualitative aspects of role models and mentorship warrant additional attention. Research could focus on identifying the qualities and influences that make mentorship relationships effective in supporting URiM students’ PIF. Tracking the long-term impact of these interventions on PIF is another important avenue for investigation. Longitudinal studies following URiM individuals throughout their medical education and early careers could offer valuable insights into how such interventions shape their professional trajectories.

Finally, a comparative analysis across countries with diverse healthcare and educational systems could identify both universal challenges faced by URiM students and culturally specific solutions. These research directions will help expand our understanding of the factors influencing URiM students’ PIF and the role that institutional practices and interventions play in shaping their experiences. Table 2 outlines these recommendations for further exploration.

**Table 2:** Recommendations for further research

Avenue for further research	Description
Practical incorporation of interventions	Assessing the feasibility, challenges, and effectiveness of implementing proposed interventions within medical institutions to guide evidence-based strategies for fostering inclusivity.
Recruitment and hiring procedures	Exploring the inclusivity of recruitment and hiring processes within medical institutions to ensure diverse representation and equitable opportunities for URiM groups.
Qualitative aspects of role models and mentorship	Examining the qualitative aspects of role modeling and mentorship relationships to understand the effective qualities and influences that contribute to positive PIF for URiM groups.
Tracking long-term impact of interventions on PIF	Following URiM individuals longitudinally throughout their medical education and early careers to gain insights into the long-term impact of interventions on PIF and career trajectories.
Comparative analysis across countries	Comparing experiences and interventions across countries with diverse healthcare and educational systems to identify universal challenges and culturally specific solutions.

## Conclusions

This systematic scoping review examined recent literature to discover the challenges faced by URiM groups in their PIF, along with the proposed interventions to address these issues within Dutch medical schools. The identified challenges encompassed the absence of role models with similar identities, ongoing microaggressions, and the pressure to assimilate into the majority culture. The literature has proposed several interventions for medical schools to address these challenges, such as recruiting more URiM professionals at the institutional level, implementing professionalism modules for medical students where URiM physicians share narratives of lived experiences, and implementing the I-CA^2^R^2^E framework to foster inclusivity and open discussion. This study contributes valuable insights that can improve medical education practices, fostering a more supportive and equitable learning environment for all students. Nonetheless, further research is recommended to enhance our comprehension of this complex topic.

## Supplementary Information

Appendix A

Appendix B

## Data Availability

The datasets used and/or analysed during the current study are available from the corresponding author on reasonable request.

## Abbreviations

PIF: Professional identity formation
URiM: Underrepresented in medicine
DEI: Diversity, equity and inclusion
PRISMA: Preferred reporting items for systematic reviews and meta-analyses
ICA^2^R^2^E: Individual, connection, acknowledge and address, reflection, role modeling, exchange

## Footnotes

1 The term ‘non-Western migrant background’ is commonly used in Dutch society to describe individuals with at least one parent born in a ‘non-Western’ country, while ‘Western migrant background’ refers to those with at least one parent born in a ‘Western’ country. According to Statistics Netherlands (CBS), individuals with a ‘non-Western migrant background’ originate from countries in Africa, South America, or Asia (excluding Indonesia and Japan), or from Turkey. Conversely, a ‘Western migrant background’ includes individuals from Europe (excluding Turkey), North America, Oceania, Indonesia, and Japan. Due to their socioeconomic and cultural position, people from Indonesia and Japan residing in the Netherlands are considered as having a ‘western’ migration background. The distinction between Western- and non-Western migrant backgrounds reflects perceived and generalized sociocultural and socioeconomic differences, rather than merely reflecting objective geographical locations [52]. 
