# Supplementary material for: Professional identity formation for underrepresented groups in medicine: challenges and interventions for Dutch medical schools: a systematic scoping review
Source: BMC Med Educ. 2025 Dec 26;25:1715. doi: 10.1186/s12909-025-07811-6 (PMC12746619; doi:10.1186/s12909-025-07811-6)
Supplement: Supplementary file 1 — Appendix A [file 12909_2025_7811_MOESM1_ESM.docx]

Appendix A. Full Search Strategy

Search Strategies – 1 Jan 2022 – 30 November 2023

| **PUBMED** |
| --- |
| ("professional identity formation"[tiab] OR "Professionalism"[Mesh]) AND ("Education, Medical"[Mesh] OR medical[tiab] OR medicine[tiab] OR physician*[tiab] OR doctor*[tiab]) – 287 |
| **EMBASE** |
| ('professional identity formation'/exp OR ‘professional identity formation’:ti,ab OR ‘identity formation’:ti,ab OR ‘professional identity’:ti,ab OR ‘professional identities’:ti,ab) AND ('medical education'/exp OR 'clinical education'/exp OR ‘physician’/exp OR ‘doctor’/exp) – 356 |
| **SCOPUS** |
| TITLE-ABS-KEY (professional AND identity AND formation) AND ( TITLE-ABS-KEY ( medicine OR medical OR clinical OR residen* OR physician* ) - 288 |
| **ERIC** |
| “professional identity” AND ("medicine" OR "medical" OR "physician" OR physicians OR "doctor" OR "doctors" OR "resident" OR "residents") – 70 |
