# Supplementary material for: Professional identity formation for underrepresented groups in medicine: challenges and interventions for Dutch medical schools: a systematic scoping review
Source: BMC Med Educ. 2025 Dec 26;25:1715. doi: 10.1186/s12909-025-07811-6 (PMC12746619; doi:10.1186/s12909-025-07811-6)
Supplement: Supplementary file 2 — Appendix B [file 12909_2025_7811_MOESM2_ESM.docx]

| \| **Authors/Year** \| **Article title** \| **Type of study** \| **Study group** \| **Challenges** \| **Proposed solutions** \| \| --- \| --- \| --- \| --- \| --- \| --- \| \| **Frost, 2013** \| “I AM a Doctor”: Negotiating the Discourses of Standardization and Diversity in Professional Identity Construction \| Critical literature review \| Medical education \| Medical students negotiate the competing discourses of standardization and diversity in different ways, constructing varied professional identities and experiencing distinct struggles in the process. \| Educators should become intentionally involved by providing ongoing mentorship, guidance, and support as students interpret and reconcile these discourses, while encouraging the preservation of individuality. Small-group and one-on-one interactions can help faculty learn about students, facilitate explicit discussions about identity construction, and clarify professional goals. \| \| **Volpe, 2019** \| Is research on professional identity formation biased? Early insights from a scoping review and meta-synthesis \| Scoping review and qualitative meta-synthesis \| Medicine, nursing, and counseling/psychology \| Most of the studies on PIF gathered at least some demographic data such as gender, age and race, very few included these data in a robust way in their analysis and interpretation of findings. Therefore, the empirical research on PIF may suffer from its own form of sociocultural bias, thereby disadvantaging trainees from diverse populations and preserving the status quo of an historically white, male medical culture. \| The authors propose to recalibrate the lenses through which PIF is viewed, conduct qualitative inquiries of diverse learners, investigate the perceptions of women and underrepresented minorities who have experienced formal PIF curricula. \| \| **Conway-Hicks S, de Groot JM, 2019** \| Living in two worlds: becoming and being a doctor among those who identify with “not from an advantaged background. \| Interviews \| Medical students (N=8), residents (N=1), physicians (N=3) \| Hidden curriculum’s contribution to silencing markers of socioeconomic under-privilege; limited formal curriculum opportunities to discuss socio-economic difference; taboo around discussing socioeconomic backgrounds; tension of ‘living in two different worlds’; intersection of socio-economic disadvantage with ethnicity, parental education, immigration and rural backgrounds. \| Safe and inclusive pedagogical opportunities to discuss socio-economic experiences, privilige and underprivilige of medical students both in relation to professional identity development and health may result in greater cultural competence of graduating physicians.  It is necessary for educators to be aware of how medical student identification with less privileged orientations may experience traumatic responses to case material that feels personal or that other students respond to, sometimes inadvertently, with microaggressions. In this regard, trigger warnings may be used to notify students of curricula that may feel personal and be adversely experienced by medical students. \| \| **Wyatt, 2020** \| What Does Context Have to Do With Anything? A Study of Professional Identity Formation in Physician-Trainees Considered Underrepresented in Medicine \| Interviews \| Black/ African American medical students (N=14) \| Lack of mentorship; negative stereotypes requiring students to manage perceptions through identity cues and engage in racial uplift. \| Responsibility to mentor URM students should not be on URM physicians. Faculties need to develop robust training programs for non-URM physicians to learn how to mentor URM students. These programs should help non-URM physicians understand the larger sociohistorical context surrounding URM students, how students actively negotiate their professional identities as minoritized individuals, and specifically target ways in which mentors can support students in the integration of their racial and professional identities. \| \| **Wyatt, 2021** \| ‘Whispers and shadows’: A critical review of the professional identity literature with respect to minority physicians \| Meto-ethnography \| 67 qualitative PIF studies (analyzed in relation to URiM physicians) \| Factors contributing to URM physicians’ PIF have only recently begun to be studied; the field of PIF research is dominated by Eurocentric frameworks that cannot account for their unique social positions; and PIF research lacks critical lenses to study how power and domination influence URM physicians’ professional identity. \| Future PIF research should adopt critical theoretical frameworks (e.g., post-colonial or critical race theory), explicitly study race, power, and social context, and use longitudinal designs with transparent reporting of participant demographics. \| \| **Wyatt, 2021** \| “Changing the narrative”: a study on professional identity formation among Black/African American physicians in the U.S. \| Interviews \| Black/African American medical students (N=14), residents (N=10), physicians (N=17) \| Constant vigilance in predominantly White medical settings; frequent microaggressions; commitment to racial uplift and giving back to one’s community; expectation to mentor and support other URiM trainees (“minority tax”). \| Redistribute mentoring responsibilities to non-URiM faculty (“majority tax”) to support the professional development of URiM trainees; increase URiM representation in academic leadership positions; and pay greater attention to the culture of Whiteness embedded in medicine’s values, beliefs, and practices, and how this culture affects URiM physicians. \| \| **Trevino, 2021** \| Professional identity formation for underrepresented in medicine learners \| Narrative review \| Medical students, residents, and fellows (UIM learners) \| UIM learners encounter challenges such as microaggressions, the minority tax, lack of mentorship, and barriers to promotion, each of which have previously been cited in the literature as contributors to the attrition of UIM faculty in academic medicine and the dissatisfaction of trainees in academia. \| Institutions should explicitly support professional identity formation through inclusive curricula, faculty development, and acknowledgment of systemic barriers. The I-CA²R²E framework (individual connection; create, acknowledge, and adjust; reflect and role model; and exchange) can be applied to strengthen individual learner support by creating safe spaces for discussion and addressing personal concerns. Inclusive communities of practice should be intentionally built to support UIM trainees through structured mentorship and sponsorship, while addressing the minority tax for faculty and learners. Developing meaningful and equitable assessment tools to evaluate and support PIF among UIM trainees is also recommended. \| \| **Bhatia-Lin, 2021** \| What Will You Protect? Redefining Professionalism Through the Lens of Diverse Personal Identities \| Descriptive \| First-year medical students (N=181) \| Rigid and racialized professionalism norms reflecting the historical White, cisgender male model; conflict between personal and professional identities among URiM and other marginalized students; emotional distress from covering stigmatized identities. \| The article describes the implementation of a 2-hour professionalism module during the first-year medical student orientation. The module aims to support all medical students, with a specific emphasis on addressing challenges faced by underrepresented minorities in medicine. \| \| **Mokhachane, 2022** \| Rethinking professional identity formation amidst protests and social upheaval: a journey in Africa \| Interviews \| Final year medical students (N=8) and recent medical graduates (N=5) \| Students have "layered identities" considering factors like race, gender, and ethnicity leading to transient professional identities. The South African context, influenced by ubuntu values and a history of apartheid, adds a unique sociohistorical twist to PIF. This work highlights the larger context of PIF, showing that personal, social, and cultural contexts, including events like social upheaval, shape the professional identity formation of medical students. \| Medical educators should strive to know students personally to understand the values, beliefs, and practices developed within their home communities. They should reconsider how students are socialized into the profession by minimizing aspects of the hidden curriculum that foster dissonance or apathy. Educators are encouraged to recognize that the foundations of professional identity are often established before medical school, shaped by childhood values and broader ontological systems, and to integrate social justice and advocacy into the curriculum as legitimate expressions of professionalism. \| \| **Bochatay, 2022** \| Towards equitable learning environments for medical education: Bias and the intersection of social identities \| Explorative \| Medical education \| URM students experience stereotype threat and microaggressions. URM physicians may struggle with an internal conflict of ‘double consciousness’ from belonging to the medical in-group while not fitting in with the prototypical identity of a physician. There is a lack of role models for URM physicians. \| To mitigate bias across all identities, three main sets of strategies can be adopted. These strategies include equipping individuals with skills to reflect upon their own and others' social identities; fostering in-group cohesion in ways that recognize intersecting social identities and challenges stereotypes through mentorship; and addressing intergroup boundaries through promotion of allyship, team reflexivity and conflict management. \| \| **Mount, 2022** \| A Critical Review of Professional Identity Formation Interventions in Medical Education \| Critical literature review \| Medical education \| Most PIF interventions rely on reflective writing and narrative reflection, reinforcing an individualist view that positions identity as a personal, internal process. This emphasis overlooks the social, cultural, and institutional contexts shaping PIF, potentially placing structural problems of medicine on individual learners. Intersectionality and systemic power relations are rarely addressed. \| Future education and research into PIF should account for theoretical preferences and the impact of these preferences. \| \| **Wyatt, 2022** \| A comparison of professional identity experiences among minoritized medical professionals \| Interviews \| PA students and practicing PAs (N=45), medical students and physicians (N=41) \| Both groups reported ongoing microaggressions and discrimination from patients and peers, and a shared commitment to community uplift. However, while minoritized PAs felt able to bring their whole selves to the profession, Black/African American physicians described a “splintered” professional identity, constantly monitoring and managing others’ perceptions to fit into the medical culture. This reflects social identity threat and the profession’s emphasis on standardization and individualism. \| Address how the culture and training of physicians contribute to identity threat and exclusion, and foster environments that enable minoritized trainees to integrate their full identities. \| \| **Malhotra, 2023** \| A Critical Appraisal of Educational Theory to Examine HBCU and Black Students' Professional Identity Formation \| Explorative \| Black Students at HBCU \| N/A \| Educational frameworks such as Tinto’s theory of student retention, Arroyo and Gasman’s HBCU educational framework, and Banks’ theory of multicultural education can be applied to promote student growth, psychological safety, and a sense of belonging in minoritized student populations. Tinto’s model highlights the importance of belonging for persistence and graduation, while Banks’ framework emphasizes integrating African American “lived experiences” and equity pedagogy into the curriculum to reflect students’ racial and cultural identities. \| \| **Spaans, 2023** \| ‘A role model is like a mosaic’: reimagining URiM students’ role models in medical school \| Interviews \| Physicians (N=10) \| Racially and ethnically underrepresented medical students are less likely to identify role models during medical school. \| Policy makers in medical schools should acknowledge that the concept of role models is not perceived unequivocally positive and can even be met with students’ resistance. Additionally, we should acknowledge the sensitivity around representative role models for URiM students as this puts the emphasis on their ethnicity, while they often work very hard just to break away from that stigma and do not wish to be addressed as an ethnic minority. \| \| **Wooten, 2023** \| Exploring the Professional Identity Formation of Racial/Ethnic Minoritized Physician Assistants \| Interviews \| Minoritized PA students and minoritized physician assistants (N=45) \| Respondents experienced microaggressions from fellow students, faculty, guest lecturers, administrators, preceptors, and other providers that affected their development as PAs. Taken together, participants' descriptions of microaggressions during both didactic and clinical training were a constant reminder of how the cultural norms created by the institution of medicine often stereotype and/or constrain the behavior of minoritized PAs. They also felt pressure to perform well to ensure they were viewed favorably and that others in their community would continue to have opportunities if they desired to move into the PA profession. \| Minoritized PAs need more support than what is currently being offered. Waiting for racially minoritized individuals to express interest in the PA profession is not enough; schools should intentionally reach out to these communities and bring them into their PA program. More support is needed to ensure that PAs who are already in the profession feel safe. Academic institutions that offer PA training need to create environments to help their trainees grow academically and professionally as well as provide them with psychological safety. Participants indicated that both mentorship and an increase in racially minoritized faculty in their programs would be useful toward continuing their development as a PA. Therefore, recruiting racially and ethnically minoritized individuals is critical to the success of the profession in meeting the population's unique health needs. \| \| **Thomas, 2023** \| The Intersection of Professional Identity Formation, Bias, and Marginalized Identities \| Integrative review \| Marginalized students in the medical field \| Students who hold marginalized identities in society can have a psychosocial experience termed “double consciousness” in which they experience themselves through their own consciousness (influenced and fostered by their minoritized culture) and through their awareness of the perceptions of members of the dominant culture around them. \| The I-CA^2^R^2^E (individual connection; create, acknowledge, and adjust; reflect and role model; and exchange) may be a suitable starting point for students from marginalized groups. \| \| **Madzia, 2023** \| “To be professional, it isn't necessarily our full selves”: How medical students with minoritized identities manage tensions between medical professionalism and their own professional identities \| Interviews \| Medical students (N=49) \| Medical students choose specialties that align with perceived professionalism standards and their social identities, construct their social identities to fit these standards, withhold aspects of their identity that conflict with them, and at times resist conformity to perceived professionalism standards. \| N/A \| \| **Maristany, 2023** \| The Problem and Power of Professionalism: A Critical Analysis of Medical Students’ and Residents’ Perspectives and Experiences of Professionalism \| Interviews \| Medical students (N=31) and senior residents (N=18) \| The current standards of medical professionalism are steeped in the historical image of a physician. Marginalized groups experience more scrutiny of their professionalism and pressure to assimilate into majority culture. \| Addressing these disparities will require change on the micro-level (teachers understanding how identity affects their standards and assessments of professionalism) and macro-level (institutions reevaluating professionalism standards and who guides professionalism remediation). \| \| **Bullock, 2023** \| ‘Yourself in all your forms’: A grounded theory exploration of identity safety in medical students \| Interviews \| Medical students (N=16) \| Participants described a variety of identity threats such as unwelcoming learning environments, feeling pressure to change one's behavior to fit in, broader sociopolitical threats and instances lacking identity safety. \| The study explored the concept of identity safety in medical education, describing how learners can exist as their authentic selves without self-monitoring. Identity safety was supported when students could leverage their identities to serve patients, when supervisors and peers upheld their personhood, and when a sense of belonging was fostered through representation and inclusion. The authors emphasized that both marginalized and majority group members share responsibility for promoting identity-safe and culturally sustaining learning environments. \| \| **Kim, 2023** \| Professional Identity Formation in Medical Education: Some Virtue-Based Insights \| Theoretical study \| Medical students \| Learners often do not feel safe in clinical settings to express a concern or ask a question, citing well-documented fears of retaliation, breaking unspoken rules, or damaging team relationships. \| Support the aspirational moral agency of learners as they engage their professionalization in relation to role models, professional norms, and the clinical learning environment. Engage students in ethical discussions about how a good doctor would act or feel in specific circumstances, and incorporate virtue-based PIF into medical education by recognizing that the excellences of a physician are embodied in learners’ lived clinical experiences and by providing ongoing, structured opportunities for humanistic and ethical reflection and guidance. \| \| **Teo, 2023** \| Assessing professional identity formation (PIF) amongst medical students in Oncology and Palliative Medicine postings: a SEBA guided scoping review \| Systematic review \| Medical students \| Medical schools show differing attention to aspects of the PIF process, which impairs evaluations, jeopardises timely and appropriate support for students, and hinders effective implementation of PIF assessments. There are also significant inconsistencies in current approaches to assessing self-concepts of identity and a lack of faculty training and support to provide timely and personalised feedback. \| The authors propose the Krishna–Pisupati model, which combines current theories and concepts of PIF to provide a holistic perspective on identity development, highlighting how clinical experiences shape students’ personhood and professional identity. Recommended strategies include implementing e-portfolios, guided reflection, mentoring, and faculty training, and using general and personalised micro-competencies to direct timely and appropriate support within a safe and structured learning environment. \| \| **Nemiroff, 2024** \| Moral injury and the hidden curriculum in medical school: comparing the experiences of students underrepresented in medicine (URMs) and non-URMs \| Interviews \| Underrepresented students in medicine (URM) (N=13) and non-URM (N=21) \| Both groups witnessed disparagement and mistreatment of patients within the hidden curriculum; URM students reported more visceral moral injury and were more likely to resist these norms. \| To address the differential moral injury that URMs experience and to improve institutional culture, participants suggested two solutions: diversifying recruitment practices and targeting the HC by emphasizing empathic clinical interactions. \| |
| --- | --- | --- | --- | --- | --- | --- | --- | --- | --- | --- | --- | --- | --- | --- | --- | --- | --- | --- | --- | --- | --- | --- | --- | --- | --- | --- | --- | --- | --- | --- | --- | --- | --- | --- | --- | --- | --- | --- | --- | --- | --- | --- | --- | --- | --- | --- | --- | --- | --- | --- | --- | --- | --- | --- | --- | --- | --- | --- | --- | --- | --- | --- | --- | --- | --- | --- | --- | --- | --- | --- | --- | --- | --- | --- | --- | --- | --- | --- | --- | --- | --- | --- | --- | --- | --- | --- | --- | --- | --- | --- | --- | --- | --- | --- | --- | --- | --- | --- | --- | --- | --- | --- | --- | --- | --- | --- | --- | --- | --- | --- | --- | --- | --- | --- | --- | --- | --- | --- | --- | --- | --- | --- | --- | --- | --- | --- | --- | --- | --- | --- | --- | --- | --- | --- | --- | --- | --- | --- |
